# A Randomized Trial of the *Little by Little* CD-ROM: Demonstrated Effectiveness in Increasing Fruit and Vegetable Intake in a Low-income Population

**Published:** 2004-06-15

**Authors:** Gladys Block, Patricia Wakimoto, Rochelle Mandel, Diane Metz, Mary L. Fujii, Nancy Feldman, Barbara Sutherland

**Affiliations:** School of Public Health, University of California; University of California, Berkeley, School of Public Health, Berkeley, Calif; University of California, Berkeley, School of Public Health, Berkeley, Calif; Solano County Cooperative Extension, University of California, Davis, Davis, Calif; University of California Cooperative Extension, Contra Costa County, Pleasant Hill, Calif; California Expanded Food and Nutrition Education Program, University of California, Davis, Davis, Calif; California Expanded Food and Nutrition Education Program, University of California, Davis, Davis, Calif

## Abstract

**Introduction:**

Research indicates that low fruit and vegetable intake is a risk factor for many chronic diseases. Despite large-scale education campaigns, the great majority of Americans do not consume recommended levels. We tested the ability of a single brief interactive experience of the *Little by Little* CD-ROM to increase fruit and vegetable intake in low-income women.

**Methods:**

A randomized placebo-controlled, parallel-group trial included 481 low-income, female participants: mean age 50.1 years, 48.4% African American, 51.6% non-Hispanic white, and 92.5% below 185% of the federally designated poverty level. Participants received one of three conditions: 1) a one-time experience with the *Little by Little* CD-ROM, 2) the *Little by Little* CD-ROM plus two reminder telephone calls, or 3) a stress management CD-ROM (control condition). We assessed baseline and follow-up dietary intake with a modified 24-hour recall.

**Results:**

Two months after the one-time experience with the CD-ROMs, both intervention groups reported significantly higher intakes of fruits and vegetables than the control group. The *Little by Little* group with reminder calls increased daily intake by 1.32 fruits/vegetables, an 86% greater increase than the control group (*P* = .016). The *Little by Little* group without reminder calls increased daily intake by 1.20 fruits/vegetables, a 69% greater increase than the control group (*P* = .052). Significantly greater movement in Stage of Readiness for Change also occurred in the *Little by Little* groups compared with the control group.

**Conclusion:**

The *Little by Little* CD-ROM may be useful in public health and clinical situations to increase fruit and vegetable intake.

## Introduction

Research overwhelmingly implicates low intakes of fruits and vegetables as factors influencing the prevalence of several chronic diseases and poor health (1-5). U.S. dietary goals recommend five to nine servings daily. Unfortunately, the average number of daily servings of fruits and vegetables consumed by men and women is fewer than four, and only about one fourth of adults report consuming five or more servings (6,7). According to *Healthy People 2010*, only 28% of Americans eat at least two daily servings of fruit; only three percent of persons consume “at least three daily servings [of vegetables], with at least one third being dark green or deep yellow” (8). Large-scale campaigns such as the 5 A Day program have increased knowledge and awareness for many Americans, but changes in actual dietary habits are small: average daily fruit and vegetable intake increased from a baseline of 3.75 servings to 3.98 servings between 1991 and 1997 (6). Some interventions have been successful, but they are typically costly and require staff-intensive approaches involving multiple in-person sessions with medical or nutrition professionals (9-11). Such interventions are not feasible in clinical or public health settings.

With the heightened focus on nutrition and its role in prevention of chronic disease, it is imperative to develop cost-effective interventions that can affect larger segments of the population.

Here we describe a randomized controlled parallel-group intervention with a brief, computer-based nutrition behavior-change program. *Little by Little*, an intervention program developed by one of the authors (GB), included a brief assessment of fruit and vegetable intake as well as messages and tips to increase intake (12,13). The study population consisted of low-income African American and white women, mean age 50.1 years (SD 7.22, range 39-65). We tested the hypotheses that subjects would demonstrate significant improvement in fruit and vegetable intake and stage of change two months after a single brief experience with the *Little by Little* CD-ROM program in comparison with a control CD-ROM, and that the *Little by Little* CD-ROM would have a greater effect on the group receiving reminder phone calls than the *Little by Little* group without reminder phone calls.

## Methods

### The *Little by Little* CD-ROM

Take a tour of the Little by Little CD-ROM

**Figure:**
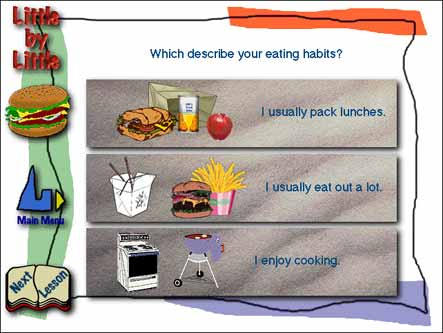
Which describes your eating habits? Audio transcription: Here we'd like to take a look at practical steps to improve what you eat. Which describe your eating habits? Text description of screen:This screen asks the user to describe his or her eating habits: Option one: I usually pack lunches. Image is of a brown-bag sandwich, an apple, and a juice box. Option two: I usually eat out a lot. Image is of a burger and fries, and a Chinese food take-away box. Option three: I enjoy cooking. Image is of a stove and a barbeque grill.

**Figure:**
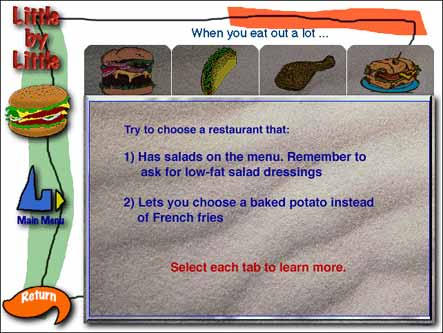
When you eat out a lot... Audio transcription: Today, with busy schedules, many of us eat out often. The problem with eating out too often is that it can be difficult to eat low-fat meals, because restaurants don't have many low-fat choices on their menus. The key is to make better choices whenever you can. If at all possible, choose a restaurant that has salads on the menu or offers a salad bar. And remember to ask for fat-free or low-fat salad dressings. It's also a good idea to know which restaurants allow you to substitute a baked potato or steamed vegetables for French fries or chips. Here are four fast-food menus. Select the tabs for each restaurant, and learn more about making better choices when you eat out. Text description of screen: This screen offers four restaurant options: A hamburger restaurant, a Mexican restaurant, a fried chicken restaurant, and a sandwich shop. The text reads: Try to choose a restaurant that:
Has salads on the menu. Remember to ask for low-fat salad dressings.Lets you choose a baked potato instead of French fries. Select each tab to learn more.

**Figure:**
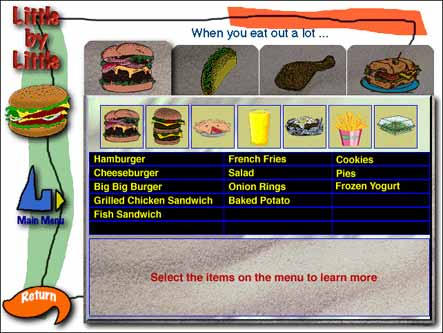
When you eat out a lot... Audio transcription: Even at a fast-food burger restaurant, some choices are better than others. Select the items on the menu to learn more. Text description of screen: In this screen, the hamburger restaurant option has been selected, and a sample menu from the restaurant is displayed. Menu items include:

**Table:** 

Hamburger Cheeseburger Big Big Burger Grilled Chicken Sandwich Fish Sandwich	French Fries Salad Onion Rings Baked Potato	Cookies Pies Frozen Yogurt

The text reads: Select the items on the menu to learn more.

**Figure:**
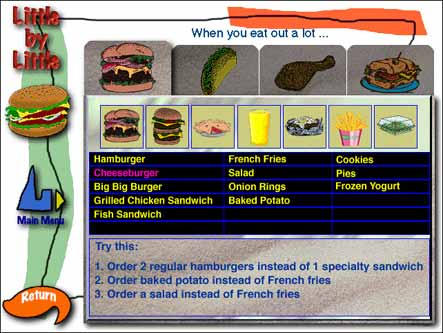
When you eat out a lot... Audio transcription: Cheese adds a lot of fat to your burger. To lower the fat in your meal, order your burgers without cheese. Text description of screen: In this screen the burrito menu item has been selected. The text reads: Try this:
Order 2 regular hamburgers instead of 1 specialty sandwichOrder baked potato instead of French friesOrder a salad instead of French fries Select each tab to learn more.

**Figure:**
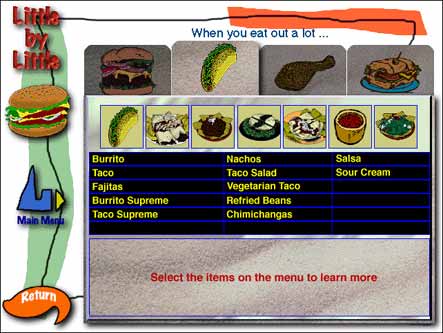
When you eat out a lot... Audio transcription: When eating out at a Mexican restaurant, how can you make better menu choices? Select each item on the menu to learn more. Text description of screen: In this screen the Mexican restaurant option has been selected, and a sample menu from the restaurant is displayed. Menu items include:

**Table:** 

Burrito Taco Fajitas Burrito Supreme Taco Supreme	Nachos Taco Salad Vegetarian Taco Refried Beans Chimichangas	Salsa Sour Cream

The text reads: Select the items on the menu to learn more.

**Figure:**
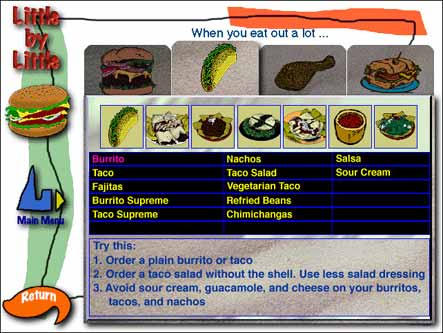
When you eat out a lot... Audio transcription: In a Mexican restaurant, plain burritos can be a good choice. Ask for low-fat cheese or one half the normal amount of cheese. Text description of screen: In this screen the burrito menu item has been selected. The text reads: Try this:
Order a plain taco or burritoOrder a taco salad without the shell. Use less salad dressingAvoid sour cream, guacamole, and cheese on your burritos, tacos, and nachos

The characteristics of the CD-ROM program are described in detail elsewhere (13). *Little by Little* was developed with funding from the Food and Nutrition Service of the U.S. Department of Agriculture (USDA). The purposes of the program are to assist the user to increase intake of fruits and vegetables and decrease intake of fat. We used only the fruit and vegetable module for this study. The objective of the program was to help participants move in small steps in the right direction toward increasing fruit and vegetable consumption. The program was based on several principles: screening and feedback, flexibility, and goal setting. First, the program included dietary screening of participants with a 10-item questionnaire on usual intake of fruits and vegetables (14) and immediate feedback to make the need for change individually relevant. Second, the program was designed to be flexible so that participants could choose topics of interest to them. Modules included suggestions for specific situations, such as when eating out, packing lunch, or cooking at home, and suggestions for barriers such as time and cost constraints. The suggestions focused on increasing the times and situations in the day when fruits and vegetables could be included, rather than on increasing servings or serving sizes. Third, the program included goal setting and individual commitment. To facilitate goal setting, the program suggested several goals to work toward, guided by options that the participant had chosen throughout the program, and the participant was asked to choose one or two of them.

### Study design and recruitment

The University of California Berkeley Committee for Protection of Human Subjects approved the research, and eligible participants provided informed consent. Data collection took place during a nine-month period, from February through October 2002. We gave a $25.00 gift card from a local store to each participant as an incentive to complete the study. We recruited study participants in collaboration with nutrition advisors and staff of the California Expanded Food & Nutrition Education Program in Contra Costa and Stanislaus counties, and the University of California Cooperative Extension and Food Stamps Program in Solano County. Staff included two part-time interviewers at each site, one African American, and one non-Hispanic white. Methods of recruitment included posted flyers inviting participation of study subjects and presentations to classes sponsored by organizations and agencies serving low-income clients. Recruitment sites included community-based organizations and selected programs that provided services to the target population, such as the Welfare to Work program, Food Stamps program, and other social services programs. Recruitment efforts also targeted lower-paid staff at day care centers, Head Start programs, social service agencies, and county offices.

To be eligible for the study, the individual had to be female, African American or non-Hispanic white, midlife (defined as 40 to 65 years of age), and low-income as reflected by their participation in the above programs serving low-income persons.

Participants were interviewed at baseline, primarily at county offices, and were randomized to one of three intervention groups, using a computer-generated randomization scheme. Group 1 received a brief, self-guided 15- to 20-minute interactive experience with a computer-based program, *Little by Little*. Group 2 received the same interactive experience with the *Little by Little* program, plus two reminder telephone calls over the next two months. Group 3 received a computer-based, interactive experience with a non-dietary CD-ROM program, *Stress Management: A Healthy Balance* (Learning Multisystems, Madison, Wis), which also lasted 15 to 20 minutes. Blocked stratified randomization on race was carried out separately in each county to ensure balance on those factors across the treatment groups. For groups 1 and 2, the intervention included goal setting as well as take-home handouts and supporting materials reinforcing the suggestions and tips from the *Little by Little* program.

For the intervention with the reminder calls, two brief telephone calls were made to the participant within the subsequent two-month period. A script for these calls was provided. The interviewer simply asked, “Do you remember the personal goal you set when you did the computer program a few weeks ago? And what was it? How have you done? If you had a hard time, what was the reason?”

### Data collection

Data were collected on age; level of education; race; income; number of persons in household; and food insecurity. Knowledge and attitudes about diet and health were also assessed, and participants were asked about obstacles and barriers to eating fruits and vegetables. Stage of Readiness for Change was assessed at baseline and follow-up and categorized in four stages, corresponding to Precontemplation (“No” to “Have you ever thought about eating more fruits and vegetables?”), Contemplation (“Yes” to that question), Preparation (“Planning to increase fruits and vegetables in the next one to two months”), and Action/Maintenance (“Currently trying to eat more fruits and vegetables”) (15,16).

Fruit and vegetable intake was assessed at baseline and follow-up using a modification of the California Dietary Practices Survey (7). The number of fruits, vegetables, and juices consumed on the previous day was obtained through a modified 24-hour recall: for each meal, the respondent was asked whether the meal was eaten and whether any fruits, vegetables, or juices were consumed. If consumed, each fruit, vegetable, or juice was recorded. For each of four meals (morning, midday, evening, snack), up to seven items could be recorded. Portion-size pictures of ¼-, ½-, 1-, and 2-cup servings were used as references to assist participants in estimating amounts consumed. Actual drinking glasses, with 4-, 8-, 12-, and 16-ounce capacities, were used to help estimate amounts of juices consumed.

### Data analysis

Linear regression and correlation techniques were used for continuous data, and classification and chi-square evaluation were used for categorical data. Baseline comparisons between groups were examined using chi-square tests for categorical data and analysis of variance for continuous data. To evaluate the effectiveness of the intervention, analysis of covariance was used, with change score as the dependent variable and baseline level as a covariate. Potential effect modification of treatment effect by race; site; education; income; and other variables in the dataset was examined. Potential confounding by those factors was also examined. The only significant covariate was site (Contra Costa, Solano, or Stanislaus County), which was included in all models.

Poverty guidelines for each household size were obtained from the U.S. government for the year 2002 (17). Poverty Index Ratio was calculated as the ratio of the reported income to the poverty guideline for that individual, based on household size. The midpoint of the income category was used to represent income. For persons who indicated that their annual income was less than $10,000, the midpoint value was set at $8,860, the poverty level for a single person. If any single persons in that income category (n = 32) had income less than $8,860, they would be calculated as having an income at the poverty level, when in fact they were below the poverty line.

Missing values for income (n = 11) and weight (n = 15) were replaced with the median value. Because of small numbers in some categories of education, the lower three categories were combined into one category for some analyses.

Two outcome variables were derived to estimate change in fruit and vegetable intake: occurrences and servings. Occurrences were simply the number of times a fruit or a vegetable was mentioned during the 24-hour recall. For example, a person who had orange juice at breakfast and orange juice at lunch would have two occurrences. Servings were calculated by applying a factor to each occurrence, based on the respondent's reported portion size for that food. For example, the USDA Food Guide Pyramid serving size for vegetables is ½ cup; if the respondent reported the smallest portion, ¼ cup, for green beans, she was credited with ½ of a serving of green beans. Treatment effectiveness was analyzed separately for change in occurrences and change in servings.

## Results

Four hundred ninety-one low income, midlife, African American and white women were enrolled and 481 (98%) completed the study. The sample included 48.4 % African American (n = 233) and 51.6% white (n = 248) women. Table 1 describes characteristics of the study population. There were no significant differences among the intervention groups in age; race; income; poverty; body mass index (BMI); baseline Stage of Readiness for Change; or baseline fruit and vegetable intake. One factor, whether or not the participant lived alone, was significantly different across treatment groups at *P* = .02. Most participants worked outside the home, and for approximately half, there were children younger than 18 years in the household. The combination of income and number of persons in the household placed two thirds of the sample below the poverty line, and more than 92% were below 185% of poverty, the cut-off for a number of federal assistance programs. Almost 75% were overweight or obese. More than 70% reported that they were currently trying to improve their fruit and vegetable intake. However, the average number of times that fruits and vegetables were reported consumed at baseline was 3.4 times per day, and only about one fourth of the sample consumed five or more servings of fruits and vegetables on the day of the survey.

### Increase in occurrences of fruits and vegetables

Change in occurrences was significantly higher in both *Little by Little* groups than in the stress-reduction group (Table 2). There were no significant interactions with race, site, education, or other factors. The *Little by Little* group with phone calls increased fruit and vegetable intake by 1.32 occurrences per day, compared with 0.71 occurrences per day in the stress-reduction group (*P* = .016). The *Little by Little* group with no telephone follow-up increased fruit and vegetable intake by 1.20 occurrences per day, also significantly greater than the stress-reduction group (*P* = .052). The *Little by Little* group with the phone calls increased fruit and vegetable intake 86% more than the stress-reduction group, and the *Little by Little* group with no telephone follow-up increased fruit and vegetable intake 69% more than the stress-reduction group. It is notable that intake of fruits and vegetables increased in the stress-reduction group as well.

### Increase in servings of fruits and vegetables

In preliminary analyses, change in servings was not significantly different across intervention groups. A significant interaction with education level was found, however, and results are presented separately by education level (Table 3). For those with a high school education or less, there was a significantly greater increase in fruits and vegetables in the *Little by Little*group with telephone reminders, compared with the stress-reduction group. In contrast, among those with an education beyond high school, neither *Little by Little* group had a significantly greater increase in fruits and vegetables than the stress-reduction group. Instead, the stress-reduction group had a substantial increase in fruit and vegetable intake (1.32 servings).

### Increase in Stage of Readiness for Change

Both *Little by Little* groups increased in Stage of Readiness for Change, but only the *Little by Little* group with telephone follow-up was statistically significant (*P* = .01) compared with the stress-reduction group (Table 4). Among individuals not already in the “currently trying” stage at baseline, 73% of individuals in the two *Little by Little* groups moved forward in stage, compared with 58% in the stress-reduction group (data not shown).

## Discussion

This study has demonstrated that the number of times that fruits and vegetables are consumed by an individual in a population of low-income women can be increased by a single experience with the *Little by Little* interactive CD-ROM. In addition, Stage of Readiness for Change was improved, and 73% of those not already at the implementation stage had some forward movement.

The U.S. Preventive Services Task Force (USPSTF) has extensively reviewed the effectiveness of interventions to improve dietary behavior (9). The analysis concluded that “moderate- or high-intensity counseling interventions, including the use of interactive health communication tools, can . . . increase intake of fruit and vegetables. Brief counseling of unselected patients by primary care providers appears to produce small changes in dietary behavior.” Two studies published since that review are consistent with the review’s conclusion: Stevens et al (18) and Steptoe et al (19) found significant improvements following either two 45-minute counseling sessions, including computer interaction (18), or two 15-minute individual counseling sessions by research nurses (19). We are not aware of any other research in which a single, brief exposure to an interactive CD-ROM, without any individual counseling, produced significant increases in fruit and vegetable intake.

It is difficult to compare effect sizes across different studies because of differences in the methods of measurement and differences in the intervals between intervention and evaluation. Among the interventions to increase fruit and vegetable intake reviewed by the USPSTF and the two more recent studies mentioned above, the interval between intervention and measurement of behavior change ranged from two to 18 months. We report here on results after a two-month interval; a one-year follow-up is in progress. For method of measurement, most studies used some form of food-frequency questionnaire, whereas we used a modified 24-hour recall, generally considered more accurate when information on absolute amount of intake by a group is desired, rather than just a ranking (20). According to the classification system used by the USPSTF (9), the effect size found in our study would be considered “medium.” Our effect size does not reach “large” primarily because the control group also had a substantial increase in fruit and vegetable intake.

The outcome variable that was most consistently affected was occurrences of eating fruits and vegetables, while servings were increased only in the less educated participants. There are a number of possible reasons for this. First, the suggestions and goals offered by the *Little by Little* program are almost exclusively aimed at increasing occurrences. Tips focused on increasing the frequency with which fruits and vegetables were chosen, such as “I will have a piece of fruit with breakfast,” rather than on portion size, such as “I will eat a larger portion of green beans.” It is also notable that among those with more than a high-school education, persons in the control (stress reduction) group increased their intake dramatically. The *Little by Little* groups increased by approximately one serving, but the more educated women within the stress-reduction group increased intake by 1.32 servings. It is possible that stress reduction, itself an important health factor, was more important in the lives of those participants.

A second possible explanation is that the baseline dietary assessment was itself an important intervention in that group. Abundant anecdotal evidence shows that simply completing a dietary questionnaire can have an effect on dietary habits in some individuals, with responses like “Wow, I never realized I ate so few fruits and vegetables.” Perhaps this was particularly true in the higher education group. As noted, the control group increased by 0.70 occurrences overall and by 1.32 occurrences in the higher education group. This alone may be sufficient justification for conducting routine dietary assessment screening as a potentially useful nutritional intervention.

Regardless of the explanation for the greater apparent effectiveness in increasing the number of occurrences, it is the opinion of one of the authors (GB) that it would be more prudent public health advice to encourage people to increase the number of occurrences, rather than focusing on the number of servings. Recommending “five to nine servings” requires people to learn the definition of a serving, and the recommendation itself probably seems unreachable to many people. (In addition, the epidemiologic literature upon which such recommendations are made has at its basis a calculation of number of times per day, not calculations involving units of measure.) Instead, what is most important is simply that fruits and vegetables show up more often in the daily diet of the population. People already know what a fruit or a vegetable is (salads count, juices count), and they can simply count the number of times they show up on the plate. However, for Asian or Hispanic populations where mixed dishes are the norm, it may be important to have an additional focus on increasing the amount of the vegetables consumed.

Behavior change is difficult, and previously only extensive, intensive interventions have been successful in changing dietary habits (9,21). The finding that a brief, one-time intervention could actually achieve dietary change is surprising, even to the authors. Why was it successful? We believe there are a number of factors. First, it should be acknowledged that midlife women are probably the one group most likely to be receptive to any dietary improvement messages (22). In addition, however, we believe that several features of the *Little by Little* program play a key role in its success: the initial dietary screening and feedback; the element of individual choice; the simplicity of the small steps suggested; and goal setting.

The first critical feature is the *Little by Little* dietary screening questionnaire that begins the program. People are unlikely to undertake change based on generalizations about what the whole population should be doing. Instead, people are more likely to respond to personalization. Research has shown that many people overestimate their fruit and vegetable intake (23), and think their own dietary intake needs no improvement (24). Consistent with Weinstein’s Precaution Adoption Process model (25), baseline evidence of personal risk behavior is an essential precursor to successful behavior change. Individuals are likely to want to change, or even to hear messages about nutrition, only if they have been shown the areas in which their own dietary intake is not up to the recommended levels.

In addition, it is possible that the simple process of asking individuals to reflect on their diets and to report on their intake is relevant, even if they are reporting that information to a computer. Physicians rarely ask patients to reflect or report on their dietary habits (8); being asked in the *Little by Little* program is evidence that “somebody” cares and considers it important. Evidence in the tobacco literature shows that if individuals are simply asked or told by a physician to stop smoking, their chances of quitting improve (26). Also probably critical is that the program responds instantly to participants with feedback and information that is directly based on information they provided. Once again, it is not generalization, but personalization.

In a related issue, the program asks participants to indicate perceived barriers to eating more fruits and vegetables, factors such as “it costs too much,” “it takes too much time,” and “the family doesn't like them.” The program offers this additional opportunity for participants to tell “someone” about their problems.

The second key factor in the success of the *Little by Little* program is the element of individual choice. The program was not designed as a type of course or set program of information, tips, and experiences to be presented and used by all participants in an identical way. Rather, the program presented a variety of options, and each participant selected only the ones in which she was interested. Participating in all program modules easily takes an hour or more, but most participants spent no more than about 15 minutes participating only in the program components they deemed most relevant. Research has shown that the ability to make individual choices enhances participation and attention (27,28) and ultimately leads to learning and behavior change.

The third critical aspect is that the changes proposed by the *Little by Little* program are easy to put into practice. The very name of the program, *Little by Little*, emphasizes ease. We cannot expect people to make wholesale changes in their behavior, and they may fear to undertake any change if they believe it will be too difficult. As participants explored different modules of *Little by Little*, they were offered easy, common-sense tips and suggestions to move them toward their dietary goal. The objective was to move them in the right direction toward increasing their fruit and vegetable intake, even if only in small increments.

A fourth critical aspect of the *Little by Little* program is goal setting. When participants had completed as many of the modules as they chose to, the program presented a list of small, easy goals based on the modules they had explored. The program asked them to choose one or two goals to work toward during the next month or so. Such goals were, “I will have one vegetarian meal each week” or “I will take a piece of fruit to work for a snack.” At the conclusion of each session, we gave participants a printed copy of their baseline dietary screening results and a copy of their chosen goals.

Another aspect that may have contributed to the program’s success is that computer screens are similar to television screens, providing a familiar, non-threatening, and credible medium to program participants. Many participants were women who had little or no experience with computers, yet they required only a few seconds of instruction on using the mouse and had no difficulty using the program independently. Rather than appearing reluctant or intimidated by the computer, participants seemed to find the experience enjoyable and to appreciate the opportunity to use a computer.

Finally, the personal contact between interviewers and participants prior to the start of the CD-ROM experience probably was influential. For the most part, interviewers were of the same ethnic group as participants, and most interviewers were also low-income. In addition, in the group that received the two reminder phone calls, the calls were made by the same interviewer who had interviewed participants at baseline and introduced them to the CD-ROM.

It is worth noting that the *Little by Little* program did not tailor the intervention to the participants’ Stage of Readiness for Change or Self-Efficacy. Rather, the program was based on behavior change and learning theory and on respect for the participants’ ability to choose input, tips, and goals consistent with her self-perceived constraints and lifestyle.

In summary, the following characteristics are key to the success of the *Little by Little* program: 1) baseline screening and feedback about the participant's current intake status; 2) flexibility, individual choice, and exploration; 3) easy, small steps; and 4) goal setting.

While we tested the *Little by Little* program among low-income persons, the program is appropriate for any English-speaking adults with access to a computer. In 2001, 60.2 million U.S. homes (56.5%) had a personal computer (29). More important, a much larger proportion of Americans, 65.6%, are computer users at some location, including worksite, public library, community center, or someone else’s house. Even among the lowest income category, those with an annual household income under $15,000, approximately 25% were computer users in 2001, and that proportion is growing at a rate of 25% per year (29). Other locations that could increase computer access for low-income persons include WIC (Special Supplemental Nutrition Program for Women, Infants, and Children) and Food Stamp offices, employment offices, and senior centers.

The components of the *Little by Little* program translate easily into many public health settings. In its original testing phase, the program was administered in libraries and senior centers (13). Equally important, the components could be integrated into clinical practice. The USPSTF concluded that “interventions using self-help materials and interactive communications . . . along with brief provider advice produced medium changes and appeared to be relatively feasible for use in primary care practices.” The brief screening questionnaire exists in both computerized and one-page, paper-and-pencil form (14,30), is self-administered, and could become part of the patient record. Combined with a 30-second admonition by the provider that “Your diet is too low in fruits and vegetables; you need to eat more of them,” the screening questionnaire alone could have some effectiveness, based on the experience with smoking cessation. This study suggests that real dietary change could be achieved if health care providers followed up the screening by loaning the *Little by Little* CD-ROM to patients or by making it available in their waiting rooms.

The *Little by Little* CD-ROM may be obtained from the School of Public Health, University of California, Berkeley, by sending an e-mail to LittlebyLittleUC@netscape.net

## Figures and Tables

**Table 1:** Sample Characteristics, *Little by Little* Effectiveness Study, Northern Calif, 2002

	**Whole Sample** **N (%)**	**Intervention Group**			***P* a**

		***Little by Little* CD-ROM with reminder phone calls** **n = 162**	***Little by Little* CD-ROM without reminder phone calls** **n = 160**	**Stress- reduction** **CD-ROM** **n = 159**	

**Race/ethnicity**					.83

African American	233 (48.4)	76	77	80	
White non-Hispanic	248 (51.6)	86	83	79	

**Income**					.51

< $10,000	112 (23.3)	36	34	42	
$10,000–$15,000	46 (9.6)	17	13	16	
$15,000–$20,000	41 (8.5)	13	17	11	
$20,000–$25,000	37 (7.7)	14	14	9	
$25,000–$35,000	82 (17.1)	31	22	29	
$35,000–$50,000	56 (11.6)	16	22	18	
$50,000–$65,000	32 (6.7)	12	6	14	
> $65,000	75 (15.6)	23	32	20	

**Poverty Index Ratio category**					.93

Below poverty	327 (68.0)	109	113	105	
100–185% of poverty	118 (24.5)	41	36	41	
Above 185% of poverty	36 (7.5)	12	11	13	

**Food insufficient^b^**					.46

Yes	107 (33.7)	32	35	40	
No	210 (66.0)	78	65	67	
Missing	1 (0.3)	1	0	0	

**Participating in Food Stamps program^b^**					.40

Yes	66 (20.8)	18	22	26	
No	251 (78.9)	92	78	81	
Missing	1 (0.3)	1	0	0	

**Live alone**					.02

Yes	87 (18.1)	38	19	30	
No	394 (81.9)	124	141	129	

**Education **					.44

Elementary school only	3 (0.6)	1	1	1	
Junior high only	10 (2.1)	2	4	4	
High school graduate	149 (31.0)	58	39	52	
More than high school^c^	319 (66.3)	101	116	102	

**Work outside the home **					.30

Yes	311 (64.7)	98	110	103	
No	170 (35.4)	64	50	56	

**Body Mass Index**					.37

Underweight (< 18.5)	6 (1.3)	0	4	2	
Normal (18.5–24.9)	118 (24.5)	40	35	43	
Overweight (25–29.9)	140 (29.1)	51	52	37	
Obese (30–34.9)	96 (20.0)	30	30	36	
Obese II (> 35)	121 (25.2)	41	39	41	

**Number of children in household**					.31

0	252 (52.4)	87	75	90	
1	107 (22.3)	29	42	36	
2 or more	122 (25.3)	46	43	33	

**Stage of Readiness for Change **					.86

Precontemplation	73 (15.2)	28	21	24	
Contemplation	34 (7.1)	10	12	12	
Preparation	33 (6.9)	8	13	12	
Action/maintenance	341 (70.9)	116	114	111	

**Fruit and vegetable occurrences/day**					.54

0	30 (6.2)	8	12	10	
1	74 (15.4)	23	22	29	
2	88 (18.3)	25	31	32	
3	75 (15.6)	23	28	24	
4	80 (16.6)	27	30	23	
5 or more	134 (27.9)	56	37	41	

**Mean age in years^d^** **(range)**	50.1 (39–65)	51.1 (39–64)	49.7 (39–64)	49.6 (39–65)	.18


a Significance of difference of characteristics across intervention groups, by chi square.

b Questions on food insufficiency and food stamp participation were not asked of persons with income above $35,000. Some persons with higher incomes could be food insufficient because of the number of persons that income had to support, but they are not included in this count.

c Included trade school.

d 60 participants declined to answer.

**Table 2:** Effectiveness of Intervention for Increasing Occurrences of Fruit and Vegetable Intake Among Low-Income Women, *Little by Little* Effectiveness Study, Northern Calif, 2002

**Intervention Group**	**Change in Fruit/Vegetable Occurrences^a^**	***P*** **b**
*Little by Little* CD-ROM with reminder telephone calls (n = 162)	1.32	.016
*Little by Little* CD-ROM without reminder telephone calls (n = 160)	1.20	.052
Stress-reduction CD-ROM (n = 159)	0.71	---

a Least squares mean for change, from analysis of covariance model adjusted for baseline level and site.

b Compared with stress-reduction CD-ROM intervention group.

**Table 3:** Effectiveness of Intervention for Increasing Servings of Fruits and Vegetables Among Low-Income Women, *Little by Little* Effectiveness Study, Northern Calif, 2002

**Education Level**	**Intervention Group**	**Change in Fruit/Veg Servings^a^**	***P*** **b**
High school or less	*Little by Little* CD-ROM with reminder telephone calls	1.22	.01
	*Little by Little* CD-ROM without reminder telephone calls	0.78	.13
	Stress-reduction CD-ROM	-0.04	---
More than high school	*Little by Little* CD-ROM with reminder telephone calls	0.87	.23
	*Little by Little* CD-ROM without reminder telephone calls	1.05	.46
	Stress-reduction CD-ROM	1.32	---

a Least squares mean for change, from analysis of covariance model adjusted for baseline level and site.

b Compared with stress-reduction CD-ROM intervention group.

**Table 4:** Effectiveness of Intervention for Increasing Stage of Readiness for Change Among All 481 Low-income Female Participants^a^, *Little by Little* Effectiveness Study, Northern Calif, 2002

**Intervention Group**	**Change in Stage^b^**	***P* c**
*Little by Little* CD-ROM with reminder telephone calls	0.41	.01
*Little by Little* CD-ROM without reminder telephone calls	0.31	.15
Stress-reduction CD-ROM	0.17	---

a Includes persons who were already at the top of the scale, Action/Maintenance, at baseline.

b Least squares mean for change, from analysis of covariance model adjusted for baseline level and site. One unit (1.0) equals a move of one level in the four Stage of Readiness for Change level (see Methods) (15,16).

c Compared to stress-reduction CD-ROM intervention group.
